# Special Issue “Health and Performance Through Sports at All Ages 3.0”

**DOI:** 10.3390/jfmk10020217

**Published:** 2025-06-06

**Authors:** Gianpiero Greco

**Affiliations:** Department of Translational Biomedicine and Neuroscience (DiBraiN), University of Study of Bari, 70124 Bari, Italy; gianpiero.greco@uniba.it

In recent years, the growing attention of the scientific community on the benefits of sport and physical activity for health and performance has generated a significant body of research. Sports practice at all ages now represents one of the pillars of health promotion and features in the prevention of numerous chronic diseases, as well as being a powerful tool for improving psychophysical well-being and individual performance capacity [1,2,3,4].

The aim of this Special Issue, “Health and Performance through Sports at All Ages: 3.0”, is to provide an updated and multidisciplinary overview of the effects of physical exercise and training methodologies across different sporting contexts: individual, team, and combat sports. From children to the elderly, sports science continues to demonstrate its relevance in the development of motor skills, improvement of body composition, and prevention of metabolic and musculoskeletal disorders [4,5,6,7].

Evidence suggests that the adoption of targeted training protocols, combined with appropriate nutritional strategies and effective recovery techniques, can positively influence not only athletic performance but also long-term health [8,9,10]. In particular, research has highlighted the importance of muscular strength and cardiovascular endurance as predictive markers of functional health [11,12,13].

The interaction between body composition and physical fitness in young athletes [3], performance optimization based on tactical roles in team sports [2], and the effectiveness of school-based programs aimed at improving physical capabilities [1] are just some topics that deserve further investigation. Moreover, the development of wearable technologies and functional testing has made it possible to refine performance analysis and personalize interventions [14,15,16], thereby supporting the implementation of targeted training and monitoring strategies. For example, the importance of resistance training from early developmental stages emerges as a key factor in supporting balanced neuromuscular development [17], while muscular strength is increasingly recognized as a crucial determinant of athletic performance across many disciplines [18]. Equally important is the implementation of practical and effective nutritional strategies to support training loads and recovery phases [19], along with the need to monitor and prevent overtraining conditions through validated and up-to-date tools, with a view to protecting athlete health [20].

This third edition of the *JFMK* Special Issue invited researchers and professionals in the field to contribute with original studies, systematic reviews, meta-analyses, and brief reports in order to enrich the scientific debate and translate knowledge into evidence-based practices useful in the field. Indeed, the articles included in this Special Issue have contributed to expanding knowledge in this field, providing new scientific evidence to satisfy the needs of all ages practicing physical activity and sports.

Twenty-six manuscripts were submitted for consideration to this Special Issue, and all were subject to the rigorous *JFMK* review process. In total, fifteen papers were finally accepted for publication and inclusion in this Special Issue (thirteen articles, a systematic review, and a case report). 

In contribution 1, the authors explored the role of inspiratory spirometry results as potential indicators for controlling training load in young swimmers. By analyzing parameters such as inspiratory flow and volume, they aimed to determine whether respiratory function could reflect physical exertion and recovery demands in this population of athletes. The results suggested that certain spirometry measurements could serve as non-invasive tools to monitor training intensity and support personalized load management in youth swimming programs.

In contribution 2, the authors conducted a systematic review to assess how anthropometric variables influence physical fitness and motor skill development in preschool children. The review highlighted that factors such as body mass index, height, and body composition significantly affect motor coordination and physical performance at this early age. These findings underscore the importance of considering anthropometric profiles when designing physical activity interventions aimed at improving motor skills and overall health in preschoolers.

In contribution 3, the authors investigated the cognitive effects of implementing short physical activity breaks during the workday among healthcare professionals. Specifically, they tested whether ten-minute bouts of exercise could enhance attention and executive function. The results demonstrated significant improvements in cognitive performance following activity breaks, suggesting that brief, structured physical interventions can be an effective strategy to boost mental efficiency and well-being in demanding work environments such as healthcare.

In contribution 4, the authors examined the effects of plyometric exercises performed with and without the ball on the development of explosive strength in volleyball players. Their findings indicated that incorporating the ball into plyometric training can further enhance performance outcomes, particularly in sport-specific explosive actions such as jumping and quick changes in direction.

In contribution 5, the study focused on stress levels and hormonal responses in a professional women’s basketball team. By monitoring biomarkers like cortisol and testosterone, the authors highlighted how physiological stress correlates with athletic performance, emphasizing the need for psychological and hormonal monitoring in high-level female athletes.

In contribution 6, the authors investigated the impact of a 3-month integrated neuromuscular training program on salivary brain-derived neurotrophic factor (BDNF) levels and motor skill development in children. The results showed a significant increase in both BDNF and fundamental motor skills, suggesting a strong link between physical training and neurodevelopmental health.

In contribution 7, the study analyzed how ankle mobility and foot stability influence jumping ability and landing mechanics. The findings demonstrated that a limited ankle range of motion and poor foot control can compromise jump performance and increase injury risk, reinforcing the importance of including mobility and stability exercises in training routines.

In contribution 8, the authors conducted a pilot study on cardiopulmonary capacity before and after knee surgery. The study aimed to explore the link between heart function and orthopedic procedures, revealing that certain parameters of cardiopulmonary fitness may be affected postoperatively, thus requiring targeted rehabilitation strategies.

In contribution 9, the authors compared the long-term physiological adaptations to short-interval high-intensity exercise, evaluating the effects of active versus passive recovery. The study concluded that both forms of recovery can support adaptations, but active recovery may lead to superior improvements in aerobic and anaerobic capacities over time.

In contribution 10, the study assessed the effects of the ActivaMotricidad program on executive functions and social relationships in early childhood education. The program showed positive outcomes in enhancing cognitive flexibility and interpersonal skills, underlining the value of structured physical activity for early neuropsychological and social development.

In contribution 11, the authors investigated associations between fundamental movement skills, muscular fitness, self-perception, and physical activity levels in primary school students. The results suggested that children with higher motor competence and muscular strength tend to engage more in physical activity and report better self-perception, highlighting the interconnectedness of physical and psychological well-being.

In contribution 12, the authors examined how biological age, sex, and geographic location influence outcomes in the Chilean National Sports Talent Detection System. The findings emphasized the relevance of maturational differences and contextual factors in talent identification, suggesting the need for tailored selection criteria across regions and age groups.

In contribution 13, the study focused on the Relative Age Effect (RAE) in children aged 9–11, analyzing how birthdate-related advantages influence anthropometric and physical fitness benchmarks. The authors found that relatively older children tend to outperform younger peers, calling for awareness and adjustment in youth sport classification and evaluation.

In contribution 14, the authors presented a case report evaluating the effects of an exercise program using immersive virtual reality on body composition and physical fitness. The results indicated that VR-based exercise can be an engaging and effective tool to promote physical health, especially in populations with low exercise adherence.

In contribution 15, the study explored how different combat sports disciplines—kicking, throwing, and grappling—affect muscular fitness and motor competence in children. The authors found that participation in combat sports enhances both strength and coordination, making them effective modalities for holistic physical development in youth.

In conclusion, the contributions presented in this Special Issue highlight the multifaceted role of physical activity and sports in promoting health, enhancing physical and cognitive performance, and supporting psychosocial development across all age groups. From early childhood to adulthood, targeted training programs, innovative methodologies, and personalized interventions have proven effective in improving motor competence, functional capacity, and overall well-being. The integration of new technologies, such as wearable devices and virtual reality, further expands the potential for individualized assessment and intervention. Continued research in this field is essential to deepen our understanding of the mechanisms through which physical activity influences health outcomes and to develop evidence-based strategies for sustainable, lifelong engagement in active lifestyles.

Given the great success of the third editions of this Special Issue, we have launched a fourth edition, for which we hope to receive contributions focusing on the effects of sports and physical activity on health and human performance across all age groups.
